# NY DEC Takes on Fracking

**DOI:** 10.1289/ehp.119-a513

**Published:** 2011-12-01

**Authors:** Charles W. Schmidt

**Affiliations:** Charles W. Schmidt, MS, an award-winning science writer from Portland, ME, has written for *Discover Magazine*, *Science*, and *Nature Medicine*.

New York is one of a handful of states (others include New Jersey, Maryland, and North Carolina) that have banned hydraulic fracturing, or “fracking,” pending further study and scientific review. A key element of New York’s review is the Supplemental Generic Environmental Impact Statement (SGEIS), a 1,537-page document drafted by the state’s Department of Environmental Conservation (DEC).^1^ The SGEIS was issued in September 2011 with a comment period scheduled to close December 12. No fracking permits have been approved in New York, and none will be until the SGEIS is finalized, according to Emily DeSantis, the DEC’s assistant director of public information.

DeSantis says fracking’s public health impacts were “fully considered” in the draft SGEIS. But a letter sent to New York governor Andrew Cuomo on October 5 and signed by more than 250 health and environmental professionals and groups claims otherwise.^2^ “The SGEIS contains no human health assessment at all,” says Sandra Steingraber, a distinguished scholar in environmental studies and sciences at Ithaca College. In the letter, signatories including Steingraber asked the DEC to conduct a supplemental analysis of baseline human health status in New York, a systematic identification and review of direct and indirect health effects of fracking, a cumulative health impacts analysis, and potential measures to eliminate those impacts.

“Scientists increasingly say there aren’t enough baseline data to draw firm conclusions about fracking’s health risks,” says Christopher Portier, director of the National Center for Environmental Health and Agency for Toxic Substances and Disease Registry of the Centers for Disease Control and Prevention. “But work done at various sites by state and federal authorities suggests additional research and analysis is warranted.” (Portier was not a signatory to the letter.)

Fracking is a method for liberating natural gas from shale rock deep underground. It generates wastewater polluted with heavy metals, salts, radionuclides, and other hazardous compounds leached from subsurface rock. Anecdotal reports of illness related to fracking operations abound, but they aren’t tracked systematically such that scientists can investigate links to specific exposures, says Robert Sweeney, chairman of the New York State Assembly Standing Committee on Environmental Conservation. Sweeney has called on New York’s state agencies to establish a registry for monitoring allegations of health issues.

But Jeffrey Gordon, director of public affairs with the New York State Department of Health, says such a registry isn’t necessary. “[This department] already has several ongoing health registries, such as the cancer, birth defects, and heavy metals registries, and other ways to access health data—for instance, from hospital admissions,” he says. “The state and county departments of health will investigate complaints of exposure to chemicals used in [fracking].”

“DEC’s focus is on preventing exposure,” says DeSantis in response to the October 5 letter. “If there are no pathways of exposure in the first place, then adverse health impacts cannot occur.” The DEC recommends a 2,000-foot setback between fracking operations and public water supplies and proposes that watersheds associated with unfiltered water supplies to New York City and Syracuse—in addition to wildlife management areas and primary aquifers that supply groundwater for human consumption—be off limits to drilling.^1^ State officials had no further comment on the letter or the degree to which human health concerns from fracking will be evaluated.
